# The Relationship Between Response Rate and Survival Benefits in Randomized Immunotherapy Studies

**DOI:** 10.3390/cancers17030495

**Published:** 2025-02-02

**Authors:** Aditi Jain, Justin Stebbing

**Affiliations:** 1 Edinburgh Medical School, Biomedical Sciences, The University of Edinburgh, Edinburgh EH8 9YL, UK; 2 School of Life Sciences, Anglia Ruskin University, Cambridge CB1 1PT, UK

**Keywords:** objective response rate, progression free survival, overall survival, immunotherapy, immuno-oncology, anti-PD-L1, trial

## Abstract

Understanding the relationship between the Objective Response Rate (ORR) and survival outcomes, notably Progression-Free Survival (PFS) and Overall Survival (OS), is relevant for assessing the efficacy of regimens in oncology. We evaluate the relationship between ORR, PFS and OS in immuno-oncology (IO) trials. Data from 68 clinical trials submitted to the FDA were evaluated, examining immunotherapy regimens, notably immune checkpoint inhibitors such as anti-programmed death (ligand)-1 [anti-PD-(L)1], cytotoxic T-lymphocyte-associated protein-4 (CTLA-4) inhibitors and combination therapies [e.g., IO + IO, anti-PD-L1 + chemotherapy, anti-PD-L1 + CTLA-4, anti-PD-L1 + TKI (tyrosine kinase inhibitors)]. Studies were included based on their reporting of ORR, PFS, and OS. Of the 68 clinical trials reviewed, 55 were included in the analysis. The correlation between ORR and PFS was moderate across most immunotherapy regimens, indicating that ORR can serve as a useful predictor of short-term disease control. However, the correlation between ORR and OS was weaker, especially in trials including combination therapies, indicating that ORR alone may not reliably predict long-term survival outcomes. ORR predicts PFS better in first-line treatment but declines in later lines and remains a weak OS predictor overall. Differing degrees of correlation between ORR and survival metrics, particularly across treatment lines and combinations, are observed. While ORR can serve as a surrogate marker for PFS in IO trials, its utility in predicting OS is restricted and the interpretation of the relationship between ORR and PFS or OS is a key limitation. Rather, a decline in PFS with increasing ORR may reflect trial differences rather than a direct relationship. Future analyses should adopt better methodologies to capture these dynamics and focus on improving surrogate endpoints for immunotherapy to improve clinical trial design and patient outcomes.

## Simple Summary

The relationships between the objective response rate (ORR), progression-free survival (PFS), and overall survival (OS) in immunotherapy are complex and nuanced, with significant implications for drug development, clinical trials, and regulatory decisions. While ORR remains a relevant metric, this analysis emphasizes that it should not be viewed as a standalone predictor of survival outcomes, particularly beyond first-line treatment and above certain ORR thresholds. To accurately predict patient outcomes and personalize therapy, clinicians should consider factors beyond ORR such as tumor biology, patient characteristics, treatment line and key survival metrics, including PFS and OS. This understanding underscores the need for a more comprehensive approach to oncology, potentially driving the development of more sophisticated biomarkers to guide immunotherapy decisions that optimize both response and survival outcomes, enhancing the efficacy and precision of cancer treatments.

## 1. Introduction

The introduction of immune checkpoint inhibitors (ICIs) has been transformative in oncology, often leading to long-lasting positive outcomes in various types of cancer such as melanoma, lung cancer, and renal cell carcinoma [1] (One of the primary metrics used to assess the effectiveness of these treatments in clinical trials is the objective response rate (ORR), which determines the proportion of patients whose tumors shrink or disappear after treatment [2] Response Evaluation Criteria in Solid Tumors (RECIST) were created to assess tumor response and progression [3]). However, tumors can behave differently in response to immunotherapies than to chemotherapeutic drugs, complicating their evaluation. To address this, the iRECIST guideline was developed by the RECIST working group for cancer immunotherapy trials. iRECIST enhances RECIST version 1.1 by standardizing tumor measurement, response definitions, and data requirements. This guideline is considered essential for immunotherapy trials to effectively capture unique response patterns, such as pseudo-progression, that traditional RECIST criteria may miss. However, the connection between ORR and more clinically significant long-term survival outcomes, notably progression-free survival (PFS) and overall survival (OS) is complex and controversial [4]) and we investigate this further here.

Further, while ORR has been a traditional endpoint in oncology trials, particularly in the context of cytotoxic chemotherapy, its validity as a surrogate for PFS and OS in the era of ICI is under increasing scrutiny [5] The distinct mechanisms of action of immunotherapies can lead to delayed responses and atypical patterns of tumor regression, challenging the conventional reliance on ORR as a predictor of long-term benefit [6] Moreover, discrepancies between ORR and OS have been observed in several studies, raising questions about the utility of ORR as a standalone endpoint for regulatory approval and clinical decision-making [7] The U.S. Food and Drug Administration (FDA) has approved several immunotherapy regimens based on improvements in these endpoints, with regular approval typically requiring evidence of enhanced survival or symptom relief; accelerated approvals have been granted based on significant ORR improvements [8,9].

Despite these approvals, the correlation between ORR and long-term outcomes such as PFS and OS in this setting remains uncertain. The aim of the study is to establish a clearer understanding of this relationship, as it will inform the design of future trials and the approval process for new therapies. We analyze ICI-based randomized clinical trials (*n* = 68) submitted to the FDA up until September 2024, including various immunotherapy regimens as monotherapy or combination therapy. We determine the correlation between ORR and long-term outcomes such as PFS and OS across these trials and, in doing so, clarify how reliably ORR can predict survival benefits in IO and contribute to understanding surrogate endpoints in cancer trials.

## 2. Materials and Methods

**Figure 1:**
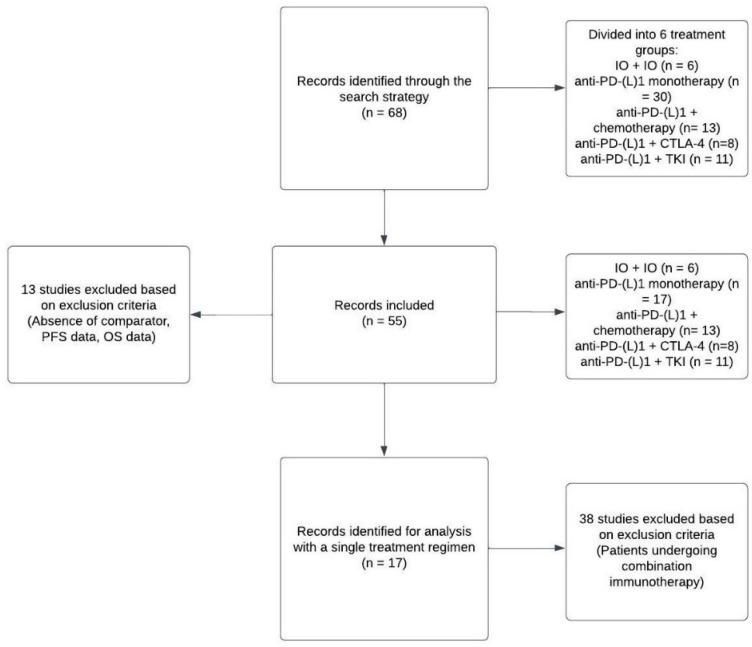
Flowchart illustrating the selection process of clinical trials for this study. NB. Flowchart created with Lucidchart.

We searched for trials that employed immunotherapy for the treatment of cancer. These trials were submitted to the FDA as initial or supplemental New Drug or Biologics License Applications until September 2024. The studies we selected were chosen to encompass a minimum of 150 cancer patients who were receiving various immunotherapy regimens, either as monotherapy or as combination therapy. The trials had a randomized, multicenter, and active-controlled design, either head-to-head or add-on. A total of 68 studies (Figure 1) were identified and categorized into five groups based on the treatment types: IO + IO (*n* = 6), anti-PD-(L)1 monotherapy (*n* = 30), anti-PD-(L)1 + chemotherapy (n = 13), anti-PD-(L)1 + CTLA-4 (n = 8), and anti-PD-(L)1 + TKI (*n* = 11). However, studies lacking a comparator and/or data for PFS and OS values were excluded. Consequently, 13 studies were excluded, leading to a final analysis comprising 55 studies (Table S1). Additionally, we examined the correlation between ORR, PFS, and OS within a particular treatment group of patients receiving a single treatment regimen, specifically anti-PD-(L)1, based on this dataset. The analysis focused on trials in which patients were treated exclusively with anti-PD-(L)1-based molecules, and only data from these regimen groups were utilized. A total of 17 trials out of the 53 trials were deemed eligible for this analysis (Figure 1).

OS was defined as the time from random assignment to death. For patients still alive at the data cutoff date, OS was recorded at the last follow-up date. PFS was defined as the time from random assignment to progression or death. Patients alive without progression were censored at their last disease assessment. PFS was primarily determined by RECIST in most trials, with variations in the versions used. ORR was defined as the proportion of patients achieving a complete or partial response according to the RECIST or WHO criteria. All analyses included the intent-to-treat population, comprising all randomly assigned patients.

The association between ORR, PFS, and OS was evaluated using linear regression models; linear regression analyses were performed on a logarithmic scale/the x axis (ORR was the log scale). We calculated the coefficient of determination (R^2^) and the associated 95% confidence intervals (CIs) from the linear regression model to measure the association between ORR, PFS, and OS. PFS and OS were presented as hazard ratios (HRs) estimated from Cox proportional hazards regression models, and ORR was presented as the odds ratios (ORs) estimated from logistic regression models. Further, we calculated the summary statistics (mean) for ORR, PFS and OS based on the reported trial outcomes. This was performed for each treatment group, as well as cumulatively for the entire dataset of ORR, PFS and OS. Summary statistics were determined to evaluate the correlations between ORR, PFS and OS across various cancer types.

## 3. Results

### 3.1. Study Selection

We searched Pubmed and the FDA website for randomized immunotherapy trials in cancer and FDA approvals, respectively, including data on ORR, PFS and OS. We identified 68 trials with immunotherapy regimens submitted until September 2024 in support of initial or supplemental New Drug or Biologics License Applications for the treatment of various cancers (Table S1). A total of 55 trials were eligible to be included in the trial-level analysis.

### 3.2. ORR Correlation with Survival Outcomes

**Figure 2:**
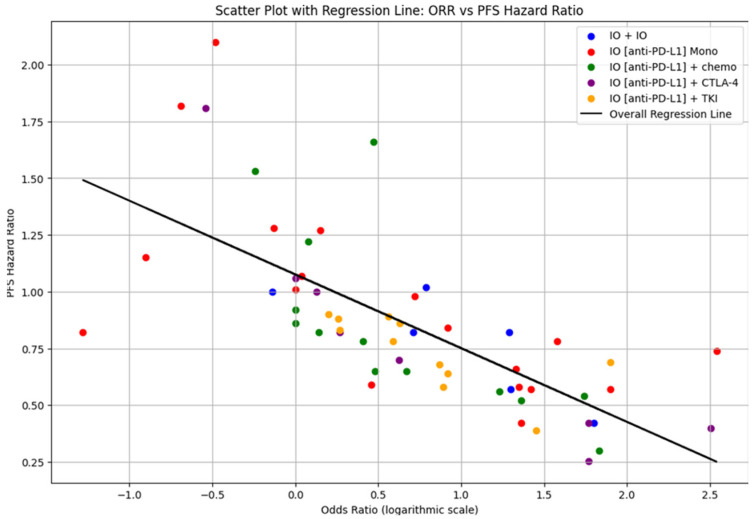
Odds ratio (ORR) and PFS hazard ratio (PFS).

The overall regression analysis of the correlation between ORR and PFS (Figure 2) from the pooled data of 55 trials shows a negative slope of −0.324, an intercept of 1.076 and an R^2^ value of 0.503. The 95% CI for the slope, ranging from −0.412 to −0.238, confirms this statistically significant negative relationship between ORR and PFS.

**Figure 3:**
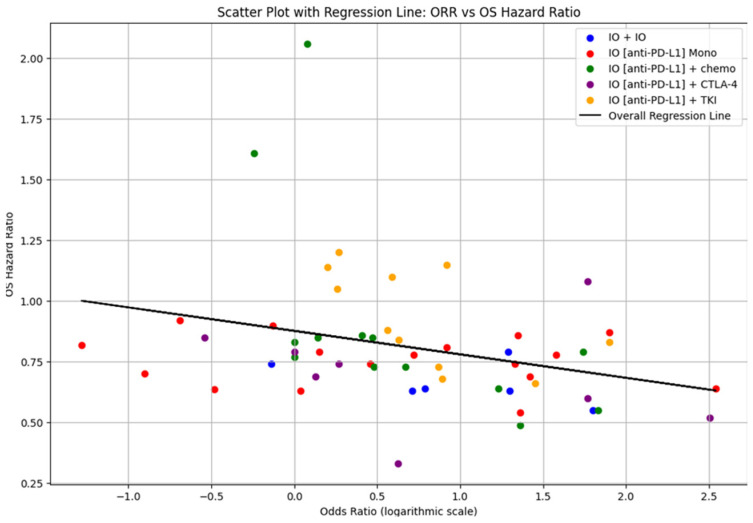
Odds ratio (ORR) and OS hazard ratio (OS).

The overall regression analysis of the correlation between ORR and OS (Figure 3) from the pooled data of 68 trials shows a negative slope of −0.125, an intercept of 0.899 and an R^2^ value of 0.157. The 95% CI for the slope, ranging from −0.412 to −0.238, confirms the statistically significant negative relationship between ORR and PFS.

### 3.3. ORR Correlation with Survival Outcomes Based on Treatment-Line

The regression analysis of the correlation between ORR and PFS based on treatment line (Figures S3 and S4) shows a negative slope of −0.360, an intercept of 1.10, an R^2^ value of 0.495 and a 95% CI (slope) of −0.481 to −0.239 for first-line treatment. For later treatments, it shows a negative slope of −0.1951, an intercept of 1.0051, an R^2^ value of 0.360 and a 95% CI (slope) of −0.338 to −0.052. The regression analysis of the correlation between ORR and OS (Figures S5 and S6) for first-line treatment indicates a negative slope of −0.110, an intercept of 0.864, an R^2^ value of 0.091 and a 95% CI (slope) of −0.226 to 0.00548009. Later treatments have a negative slope of −0.071, an intercept of 0.917, an R^2^ value of 0.138 and a 95% CI (slope) of −0.168 to 0.026.

### 3.4. Summary Statistics

Summary statistics (Table S2) show a considerable improvement in both PFS [7.80 months (experimental) vs. 5.70 months (comparator)] and OS [30.2 months vs. 16.8 months] for the IO + IO treatment group, with higher ORRs in the experimental arms [42.18% vs. 27.8%] correlating with longer survival times. The anti-PD-(L)1 monotherapy group showed modest ORR improvements [21.5% vs. 17.4%], with the OS [12.3 months vs. 9.6 months] generally improved in most trials. The improvement in PFS was small and not consistent across the treatment group [3.12 months vs. 4.24 months]. The anti-PD-(L)1 with chemotherapy treatment group revealed substantially improved ORR [48.2% vs. 32.4%] and OS [17.9 months vs. 12.6 months]. The anti-PD-(L)1 with CTLA-4 group showed significant improvements in ORR [50.0% vs. 23.0%], PFS [11.5 months vs. 5.65 months] and OS [72.1 months vs. 22.9 months]. The anti-PD-(L)1 with TKI group showed significant improvements for PFS [15.93 months vs. 9.40 months and ORR [53.1% vs. 30.8%]. Cumulatively, a strong association was observed between ORR [42.5% vs. 26.1%], PFS [8.55 months vs. 5.53 months] and OS [26.8 months vs. 17.2 months].

### 3.5. Optimal Threshold for Maximum Survival Benefit

**Figure 4:**
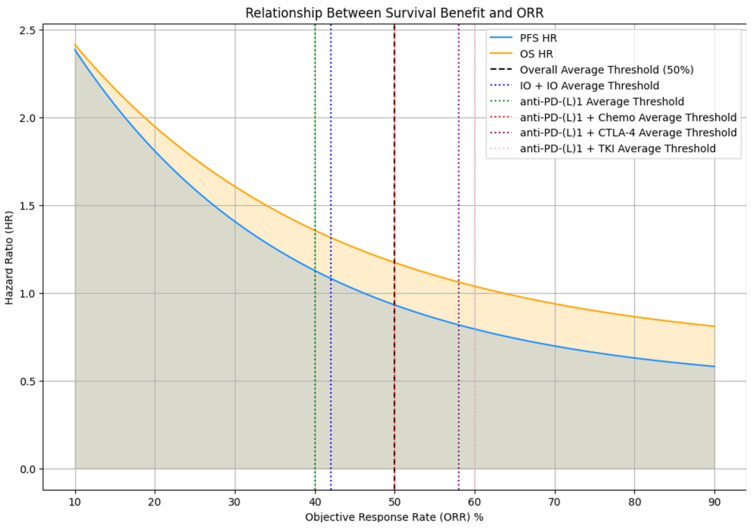
Optimal threshold for maximum survival benefit.

Among the various groups of immunotherapy treatments, a pattern is observed between ORR and survival outcomes, PFS and OS, which indicate that the improvements reduce beyond a certain ORR threshold (Table 1 and Table 2). In the IO + IO group, the ORR ranges from 35.0% to 58.0%, with the most promising outcomes being at the highest ORR levels (58.0% ORR associates with a PFS HR = 0.420 and an OS HR = 0.550). However, if the ORR drops below 42%, the survival benefit decreases, as indicated in the PFS and OS hazard ratios approaching 1.00 at an ORR of 35.0%. Similarly, in anti-PD-(L)1 monotherapy, ORR ranges from 12.0% to 43.8%, with significant PFS and OS improvements observed up to approximately 40.0% ORR (PFS HR = 0.570, OS HR = 0.690). Beyond this point, the improvements either plateau or diminish. For anti-PD-(L)1 combined with chemotherapy, the ORR varies from 29.0% to 81.5%, with the strongest outcomes linked to ORRs up to 50.0%. At higher ORRs, there is a marked reduction in improvement (OS HR = 0.860). The combination of anti-PD-(L)1 with CTLA-4 shows a comparable trend, with significant improvements at ~58.0% ORR but diminishing returns as ORR decreases to the 36.0%-40.0% range. In the anti-PD-(L)1 combined with TKI group, the ORR ranges from 22.7% to 71.0%, with ideal improvements seen up to 60.0% ORR (OS HR = 0.390), after which further increases in ORR yield less prominent improvements in survival (Figure 4).

## 4. Discussion

The relationship between ORR and crucial long-term outcomes, namely PFS and OS, in the context of immunotherapy aids in understanding the complexities of assessing cancer treatment responses [10]. The differing degrees of correlation observed across a range of immunotherapy regimens have considerable implications for both the direction of future research and clinical decision-making [4,11]. Regardless of the significant developments in immunotherapy and its assimilation into oncology, challenges remain in recognizing the most reliable surrogate endpoints for predicting the clinical benefit [5,7]. This study assesses the correlation between ORR and survival outcomes, particularly PFS and OS, across various immunotherapy regimens. These findings add to the ongoing discussion about the benefit of ORR as a surrogate marker in oncology, though the complexities of interpreting these associations are evident. To our knowledge, this is the first analysis using trial-level data to explore the correlation between ORR, PFS, and OS across numerous immunotherapy regimens in different cancers.

### 4.1. Analysis of ORR Correlation with Survival Outcomes

The overall regression analysis of the correlation between ORR and PFS shows a negative slope (0.324), which might initially suggest that as ORR increases, the PFS ratio decreases. However, this interpretation is counterintuitive, as higher response rates are generally linked to better clinical outcomes. This paradox may reflect the complex interplay of factors influencing treatment response. The intercept of 1.076 suggests that, when ORR is minimal, the PFS ratio remains positive, suggesting that other factors maintain the PFS ratio. The R^2^ value of 0.503 indicates that while ORR is a significant predictor of PFS, it is not exclusive. Nearly half the variation in PFS can be explained by other factors. The 95% CI for the slope (−0.412 to −0.238) suggests that the statistically significant negative relationship between ORR and PFS should be interpreted with caution within the broader clinical setting.

Further analysis specific to anti-PD-(L)1 therapy (Figure S1) reveals a more robust relationship between ORR and PFS. The R^2^ value of 0.840 alludes to a strong positive correlation. The 95% CI for the slope (0.120 to 0.194) confirms the statistically significant positive relationship between ORR and PFS. This finding is encouraging as it suggests that ORR could serve as a predictive marker for PFS in patients undergoing anti-PD-(L)1 therapy.

In contrast, the correlation between ORR and OS is weaker, with the slope (−0.125), revealing that as ORR increases, the OS ratio decreases marginally. This weak correlation suggests that ORR may influence OS; however, it is not a dominant factor. The intercept here (0.899) suggests that despite a minimal ORR, there is a substantial underlying survival benefit. The low R^2^ value of 0.157 reinforces that OS is affected by factors beyond ORR, such as the tumor biology, patient characteristics, and other treatment modalities. Further, while the 95% CI confirms the statistical significance of the negative slope, the weak overall correlation indicates that ORR alone is not a reliable predictor of OS outcomes.

However, the analysis specific to anti-PD-(L)1 therapy (Figure S2) reveals a moderate positive relationship between ORR and OS. The R^2^ value (0.460) suggests a moderate correlation. While a higher ORR is linked to a longer OS, other factors likely play a considerable role in determining OS outcomes. The 95% CI of the slope (0.404 to 1.59) suggests that there is a statistically significant positive relationship between ORR and OS. Although the confidence interval is relatively wide, reflecting some uncertainty in the exact magnitude of the relationship, it clearly signifies that improvements in ORR generally lead to increases in OS. This suggests that while ORR is a relevant factor in improving OS, it is not as strong a predictor as it is for PFS, and that OS outcomes are influenced by a broader array of variables.

Given that the ORR is on a logarithmic scale, the negative slope does not indicate that a higher ORR leads to worse outcomes. Instead, it suggests that proportional increases in ORR are linked to less-than-proportional improvements in PFS and OS. This could imply that at higher levels of ORR, the expected improvements in survival outcomes are not as pronounced, possibly due to diminishing returns or the complex nature of cancer progression and response to therapy. The intercepts still provide a reference point for expected outcomes when ORR is minimal. The moderate R^2^ value for PFS and the low R^2^ value for OS further underline that ORR, even on a log scale, is only one of many factors influencing survival outcomes, and that the relationships are nuanced and multifactorial.

### 4.2. Analysis of ORR Correlation with Survival Outcomes Based on Treatment-Line

The evaluation based on the treatment line reveals major differences in the relationship between ORR and survival outcomes. In first-line treatment, a moderate inverse correlation between ORR and PFS is noted, with a slope of −0.360 and an R^2^ of 0.495 (Figure S3), suggesting that approximately half of the variability in PFS is explained by ORR. This robust relationship implies that in first-line treatment, ORR is a strong predictor of PFS, though the inverse relationship opposes the standard expectation that higher ORR correlates with improved survival outcomes. Further, for OS in first-line treatment, the relationship is weaker, with a slope of −0.110 and an R^2^ of 0.091 (Figure S5), suggesting that ORR explains only a minor share of the variability in OS, and that other factors are probably more influential in understanding overall survival.

In second- and third-line treatments, the inverse relationship between ORR and PFS continues but is weaker, with a slope of −0.195 and an R^2^ of 0.360 (Figure S4). This reveals that ORR is less predictive of PFS in later lines of treatment, where other clinical factors, such as disease progression and former treatments, may play a more significant role. Likewise, the correlation between ORR and OS in later-line treatments is also weak, with a slope of −0.071 and an R^2^ of 0.138 (Figure S6), highlighting that ORR remains a poor predictor of OS in these settings. The low R^2^ values in both first-line and second-/third-line treatments for OS suggest that ORR alone cannot justify the variation in survival outcomes. Overall, the line of treatment plays an important role, with ORR being a more robust predictor of PFS in first-line treatments than in second- or third-line therapies, where other clinical factors likely have a more substantial influence.

### 4.3. Analysis of Treatment-Specific Correlation

The data indicate differences in how ORR correlates with PFS and OS across different IO regimens. For instance, in the group receiving combination immunotherapies (IO + IO), there is an evident negative correlation between ORR and PFS, indicating a moderate relationship. Conversely, the correlation with OS is weaker, suggesting that while combination immunotherapy (IO + IO) may lead to a higher ORR, this does not necessarily translate into proportionately better survival outcomes. In anti-PD-(L)1 monotherapy, the relationships appear more variable. As the correlation between ORR and PFS demonstrates a moderate but significant inverse relationship, the correlation between ORR and OS is even weaker, emphasizing the limited predictive value of ORR for overall survival in monotherapy settings.

The combination of anti-PD-(L)1 with chemotherapy shows similar trends. The correlation between ORR and PFS suggests that ORR may provide some indication of PFS outcomes in this treatment combination, but its utility in predicting OS remains limited. The data for anti-PD-(L)1 combined with CTLA-4 inhibitors reflect a more complex interaction, indicating a moderate correlation between ORR and PFS, while the OS correlation is weaker, pointing again to the limited relevance of ORR for predicting long-term survival outcomes in these patients. Lastly, for anti-PD-(L)1 combined with TKI, the data suggest a somewhat stronger correlation with PFS, while the correlation with OS remains weak.

The summary statistics reveal a strong correlation between ORR and both PFS and OS, with combination therapies having the most marked effects. The IO + IO treatment group consistently showed higher ORRs and corresponding gains in PFS and OS. The combination of anti-PD-(L)1 with chemotherapy, CTLA-4 and TKIs also demonstrates a significantly enhanced ORR and survival outcomes, highlighting the benefits of combinatorial approaches. In contrast, anti-PD-(L)1 monotherapy showed less consistent benefits, particularly for PFS, though OS improvements were still observed. Further, the experimental groups have a mean ORR of 42.5%; this is compared to 26.1% for the comparator groups in the cumulative data, which shows that there was an increased response rate for the experimental treatment groups. The experimental arms also have a mean PFS of 8.55 months, compared to 5.53 months for the comparator groups, signifying a longer time without disease progression and a better tumor response. Additionally, the former exhibit a mean OS of 26.8 months, compared to 17.2 months in the comparator arms, showing a considerable improvement in patient survival. Overall, these findings indicate that a higher ORR is generally linked with longer patient survival, that is, enhanced PFS and OS make it a valuable, though variable, predictor of clinical outcomes.

### 4.4. Defining the Optimal Threshold for Maximum Survival Benefit

These observations highlight that an ORR threshold of approximately 50% represents a significant point across most regimens, beyond which the improvements in PFS and OS begin to diminish. While some treatments may indicate improvements marginally above this threshold, the general trend suggests that an ORR beyond 50% provides declining returns in terms of survival benefits in immunotherapy.

### 4.5. Limitation of Surrogate Endpoints in Predicting Long-Term Clinical Benefit

The results of this analysis reveal the consistent correlation between PFS, OS and ORR, showing the potential risks of relying on these surrogate endpoints as the primary factor for regulatory approval. Although the ORR is commonly used to accelerate approval, especially in immuno-oncology trials, the limited correlation between long-term outcomes and the ORR stresses the need for caution. Oxnard et al. (2016) [12] supports this by highlighting that numerous drugs approved based on a high ORR via the FDA’s accelerated approval (AA) pathway have not resulted in a significant increase in OS in confirmatory trials. Additionally, this concern becomes particularly vital when considering that drugs which showed inadequate clinical benefits in post-approval trials were withdrawn. 

In this analysis, the weak correlation between OS and ORR across treatment regimens and lines further confirms the notion that ORR alone may not be a reliable predictor of OS. Moreover, the negative slope between PFS and ORR in certain settings, while statistically significant, also suggests that greater response rates do not always lead to better PFS. These findings are consistent with the concerns raised by Oxnard et al. (2016) [12]. They state that while a high ORR might support initial approvals, it is necessary to validate these endpoints through rigorous post-approval trials to confirm that they indicate real clinical benefits.

Thus, lessons from drug withdrawals should guide future regulatory approaches and research by giving priority to the validation of surrogate endpoints like ORR. Depending solely on these endpoints for approval, without validating their effect on long-term outcomes, could result in ineffective treatments being introduced into clinical practice prematurely. This emphasizes the necessity of more extensive and long-term studies to confirm that therapies not only produce a short-term response but also deliver meaningful survival benefits for patients. 

### 4.6. Study Limitations

A key limitation of our study is the interpretation of the relationship between ORR and PFS or OS. This analysis compares the odds ratio for ORR with the hazard ratios for PFS and OS, but we acknowledge that this approach does not fully capture the within-trial dynamics of treatment benefits. Specifically, within a given trial, a larger odds ratio for ORR typically corresponds to a greater treatment benefit, which is expected to translate into a lower hazards ratio for PFS or OS. Thus, the apparent decline in PFS as ORR increases that was observed in our regression analysis may reflect differences in the comparative benefit between trials, rather than a direct counterintuitive relationship. Future analyses should adopt alternative methodologies to better analyze and interpret the relationship between treatment effect metrics across trials, particularly accounting for these within-trial dynamics. 

Further, the limitations of this study include the analysis being solely based on trial-level data from clinical trials published and then submitted to the FDA. Patient-level data have not been incorporated here and, although trial-level analysis offers valuable insights into overall trends, it cannot evaluate individual patient variability, such as the variations in response based on treatment adherence, patient characteristics or disease subtypes. Further, another limitation is the heterogeneity of the included clinical trials, which involved various patient populations, cancer types, and immunotherapy regimens. There is a possibility that biases were introduced during the correlation of ORR with PFS and OS due to variations in disease biology and treatment mechanisms. The differences in follow-up durations and trial designs also complicate the interpretation of results, especially when comparing across various therapeutic strategies. Additionally, in most trials, patients changed treatment regimens due to disease progression or other reasons, and this resulted in the contamination of OS data. It is not possible to obtain “clean” OS data. We have not included bispecific molecules, and it is possible that we have unintentionally missed studies of relevance.

### 4.7. Future Research

Given the complexities observed in this analysis, exploring metrics such as the depth and duration of response in further research could offer greater insights into the treatment’s impact on disease progression. Including patient-level data could help align responses with clinical benefit. This will enable a deeper understanding of safety and treatment efficacy and aid in the identification of specific subgroups that respond differently to immunotherapy.

## 5. Conclusions

In summary, these findings question the conventional expectation that a higher ORR should directly correlate with better survival outcomes, particularly in the context of PFS and OS. While the ORR is an important indicator, it is not the sole determinant of survival outcomes in immunotherapy. This highlights the prominence of considering a broader array of factors, including molecular, biological, and clinical variables, in treatment planning and research. Based on the statistical significance of the relationships, the ORR does play a role, but its limited clinical relevance—particularly regarding OS—suggests that clinicians should not rely exclusively on the ORR when predicting patient outcomes. It is also important to note that the results from the analysis of the single treatment regimen may only apply to the patients undergoing anti-PD-(L)1 therapy, and not other types of immunotherapies.

## Figures and Tables

**Table 1:** (**A**) Summary of odds ratio (OR) and PFS hazards ratio (PFS) for IO + IO from included studies. (**B**) Summary of odds ratio (OR) and PFS hazards ratio (PFS) for IO [anti-PD-(L)1] Monotherapy from included studies. (**C**) Summary of odds ratio (OR) and PFS hazards ratio (PFS) for IO [anti-PD-(L)1] + chemotherapy from included studies. (**D**) Summary of odds ratio (OR) and PFS hazards ratio (PFS) for IO [anti-PD-(L)1] + CTLA4 from included studies. (**E**) Summary of odds ratio (OR) and PFS hazards ratio (PFS) for IO [anti-PD-(L)1] + TKI from included studies.

**(A)**	
**OR (Log Values)**	**PFS Hazard Ratio**
1.80	0.420
1.30	0.570
1.29	0.820
0.710	0.820
0.790	1.02
−0.140	1.00
**(B)**	
**OR (Log Values)**	**PFS Hazard Ratio**
1.35	0.570
1.42	0.580
0.040	0.570
0.920	1.07
−0.900	0.840
0.720	1.15
1.33	0.980
2.54	0.660
0.150	0.740
−1.28	1.27
0.460	0.590
−0.13	1.28
1.58	0.780
−0.690	1.82
−0.480	2.10
1.36	0.420
**(C)**	
**OR (Log Values)**	**PFS Hazard Ratio**
0.140	0.820
0.410	0.780
0.00	0.860
0.00	0.920
0.080	1.22
−0.240	1.53
1.36	0.520
1.23	0.560
0.480	0.650
0.670	0.650
1.83	0.300
1.74	0.540
0.470	1.66
**(D)**	
**OR (Log Values)**	**PFS Hazard Ratio**
−0.542	1.81
2.51	0.400
1.77	0.420
0.00	1.06
0.267	0.82
0.128	1.00
0.626	0.700
1.77	0.252
**(E)**	
**OR (Log Values)**	**PFS Hazard Ratio**
0.890	0.580
0.870	0.680
1.45	0.390
0.630	0.860
0.270	0.830
0.590	0.780
0.260	0.880
0.200	0.900
0.560	0.890
0.920	0.640
1.90	0.690

**Table 2:** (**A**) Summary of odds ratio (OR) and OS hazards ratio (OS) for IO + IO from included studies. (**B**) Summary of odds ratio (OR) and OS hazards ratio (PFS) for IO [anti-PD-(L)1] Monotherapy from included studies. (**C**) Summary of odds ratio (OR) and OS hazards ratio (PFS) for IO [anti-PD-(L)1] + chemotherapy from included studies. (**D**) Summary of odds ratio (OR) and OS hazards ratio (PFS) for IO [anti-PD-(L)1] + CTLA4 from included studies. (**E**) Summary of odds ratio (OR) and OS hazards ratio (PFS) for IO [anti-PD-(L)1] + TKI from included studies.

**(A)**	
**OR (Log Values)**	**OS Hazard Ratio**
1.80	0.550
1.30	0.630
1.29	0.790
0.710	0.630
0.790	0.640
−0.140	0.740
**(B)**	
**OR (Log Values)**	**OS Hazard Ratio**
1.90	0.870
1.35	0.860
1.42	0.690
0.040	0.630
0.920	0.810
−0.900	0.700
0.720	0.780
1.33	0.740
2.54	0.640
0.150	0.790
−1.28	0.820
0.460	0.740
−0.13	0.900
1.58	0.780
−0.690	0.920
−0.480	0.637
1.36	0.540
**(C)**	
**OR (Log Values)**	**OS Hazard Ratio**
0.140	0.850
0.410	0.860
0.00	0.830
0.00	0.770
0.080	2.06
−0.240	1.61
1.36	0.490
1.23	0.640
0.480	0.730
0.670	0.730
1.83	0.550
1.74	0.790
0.470	0.850
**(D)**	
**OR (Log Values)**	**OS Hazard Ratio**
−0.542	0.850
2.51	0.600
1.77	0.520
0.00	1.08
0.267	0.790
0.128	0.740
0.626	0.690
1.77	0.332
**(E)**	
**OR (Log Values)**	**OS Hazard Ratio**
0.890	0.680
0.870	0.730
1.45	0.660
0.630	0.840
0.270	1.20
0.590	1.10
0.260	1.05
0.200	1.14
0.560	0.880
0.920	1.15
1.90	0.830

## Data Availability

The data underlying this article are available in the article and in its online Supplementary Materials.

## Supplementary Materials

The following supporting information can be downloaded at: https://www.mdpi.com/article/10.3390/cancers17030495/s1, Table S1. Study characteristics for the included studies [13,14,15,16,17,18,19,20,21,22,23,24,25,26,27,28,29,30,31,32,33,34,35,36,37,38,39,40,41,42,43,44,45,46,47,48,49,50,51,52,53]; Table S2. Summary Statistics for ORR, PFS and OS; Figure S1. Correlation Between ORR and PFS in Patients Undergoing PD-(L)1 Therapy; Figure S2. Correlation Between ORR and OS in Patients Undergoing PD-(L)1 Therapy; Figure S3. First Line Treatment (PFS); Figure S4. Second- and Third-Line Treatment (PFS); Figure S5. First Line Treatment (OS); Figure S6. Second and Third Line Treatment (OS).
