# Supplementary material for: The Relationship Between Response Rate and Survival Benefits in Randomized Immunotherapy Studies
Source: Cancers (Basel). 2025 Feb 2;17(3):495. doi: 10.3390/cancers17030495 (PMC11815975; doi:10.3390/cancers17030495)
Supplement: Supplementary file 1 [file cancers-17-00495-s001.zip › cancers-3420069-supplementary(1).pdf]

cancers MDPI

SUPPLEMENTARY MATERIALS

Table S1. Study characteristics for the included studies.

Indication/Sponsor	Regimen	Comparator	Cohort	ORR (%)	PFS (months)	OS (months)
			IO + IO			
Melanoma (1L)<br>CM-067<br>BMS<br>[13]	Nivo + Ipi	Ipilimumab	NA	58% vs 19%<br>P<0.00001	11.5 m vs 2.9 m<br>p<0.0001	72.1 m vs 19.9 m<br>p<0.0001
Melanoma (1L)<br>CM-067<br>BMS<br>[13](	Nivo mono Tx	Ipilimumab	NA	45% vs 19%<br>P<0.00001	6.9 m vs 2.9 m<br>p<0.0001	26.9 m vs 19.9 m<br>p<0.0001
RCC (1L; I/P risk)<br>CM-214<br>BMS<br>[14]	Nivo + Ipi	Sunitinib	NA	42% vs 27%<br>P<0.001	11.6 m vs 8.4 m<br>p = 0.03 vs (0.009 reqd for signf.)	NR vs 25.9 m<br>p<0.0001
ESCC (1L)<br>(PD-L1 ≥ 1%)<br>CM-648<br>BMS<br>[15]	Nivo + Ipi	Chemo	NA	35% vs 20%	4.0 m vs 4.4 m<br>, p= 0.90	13.7 m vs 9.1 m<br>p=0.001
Malignant Pleural Mesothelioma (1L)<br>CM-743<br>BMS<br>[16]	Nivo + Ipi	Chemo	NA	40% vs 43%	6.8 m vs 7.2 m<br>p not reported	18.1 m vs 14.1 m<br>p=0.002
NSCLC (1L)<br>(PD-L1 ≥ 1%)<br>CM-227<br>BMS<br>[17]	Nivo + Ipi	Chemo	NA	35.9% vs 30%	5.1 m vs 5.6 m<br>p not reported	17.1 m vs 14.9 m<br>p=0.007
			PD – (L)1 Monotherapy			
Melanoma (2L+; post-Ipi)<br>KN-002 – P2<br>Merck<br>[18]	Pembro mono Tx	Chemo	N=~180 each at 2 DLs	21 – 25<br>vs 4	2.9 vs 2.7<br>p<0.001	13.4 vs 11<br>p=0.117
Melanoma (2L+; post-Ipi)<br>KN-006 – P3<br>Merck<br>[19]	Pembro mono Tx	Ipi mono Tx	N=~278 at 2 DLs	33 – 34<br>vs 12	4.1 vs 2.8<br>p=<0.001	12m OS: 68 vs 58<br>p=0.0036
Advanced Melanoma<br>CM - 067<br>BMS<br>[20]	Nivo	Ipi	316 vs 315	45 vs 19	6.9 vs 2.9	36.9 vs 19.9
Melanoma (1L)<br>All-comers<br>CM-067<br>BMS<br>[13]	Nivo mono Tx	Ipi	316 vs 315	40 vs 14	6.9 vs 2.9<br>P<0.0001	36.9 vs 19.9<br>P<0.0001
NSCLC<br>1L; PD-L1 ≥ 1%<br>KN-042<br>Merck	Pembro mono Tx	Chemo alone	637 vs 637	27.3 vs 26.5	5.4 vs 6.5	16.7 vs 12.1<br>P=0.0036

[21]						
NSCLC<br>(IO-naïve; post-chemo<br>PD-L1 ≥ 1%)<br>KN-010<br>Merck<br>[22]	Pembro mono Tx	docetaxel	346 vs 343	21.2 vs 9.6<br>P<0.001	4.0 vs 4.1<br>P=0.005	11.8 vs 8.4<br>P<0.001
HNSCC (1L)<br>PD-L1 ≥ 1%)<br>KN-048<br>Merck<br>[23]	Pembro mono Tx	Cetuximab + platinum chemo + FU	257 vs 255	19 vs 35	3.2 vs 5.0	12.3 vs 10.3<br>P=0.0171
Urothelial<br>2L+; post-plt chemo<br>KN-045<br>Merck<br>[24]	Pembro mono Tx	Chemo	270 vs 272	21.1 vs 11.4	2.1 vs 3.3<br>P=0.833	10.3 vs 7.4<br>P=0.004
mESCC<br>2L+; PD-L1 ≥ 10<br>KN-181<br>Merck<br>[25]	Pembro mono Tx	Chemo	85 vs 82	22 vs 7	3.2 vs 2.3	10.3 vs 6.7<br>P=0.0074
HCC (2L+)<br>post-sorafenib/ chemo<br>KN-394<br>Merck<br>[26]	Pembro mono Tx	Placebo	222 vs 128	13.7 vs 1.3<br>P<0.0001	2.6 vs 2.3<br>P=0.0032	13.9 vs 10<br>-=0.180
Gastric/GEJ<br>2L+ (post-plt-chemo)<br>PD-L1 ≥ 1%<br>KN-061<br>Merck<br>[27]	Pembro mono Tx	Paclitaxel	196 vs 199	16 vs 14	1.5 vs 4.1 m<br>p=0.98	9.1 vs 8.3 m<br>p=0.042
Gastric/GEJ 1L<br>PD-L1 ≥ 1 or 10<br>KN-062<br>Merck<br>[28]	Pembro mono Tx	Chemo alone	256 vs 250	15 vs 37	2.0 vs 6.4	10.6 vs 11.1
CRC<br>1L MSI-H<br>KN-177<br>Merck<br>[29]	Pembro mono Tx	Chemo alone	153 vs 154	43.8 vs 33.1	16.5 vs 8.2<br>p=0.0004	NR vs 36.7<br>p=0.0359
NPC 2L+<br>All-comers<br>KN-122<br>Merck<br>[30]	Pembro mono Tx	Chemo alone	117 vs 116	21.4 vs 23.5	4.1 vs 5.5	17.2 vs 15.2<br>p=02262
HCC 3L+<br>All-comers<br>KN-240<br>Merck<br>[31]	Pembro mono Tx	BSC	278 vs 135	18.3 vs 4.4	3 vs 2.8m<br>p=0.0209	13.9 vs 10.6<br>p=0.0238
Bladder 1L<br>All-comers<br>KN-361<br>Merck	Pembro mono Tx	Chemo alone	307 vs 352	30 vs 45	3.9 vs 7.1 m<br>p=untested	15.6 vs 14.3<br>p=untested

[32]						
Gastric/GEJ KN-063 Merck [33]	Pembro mono Tx	Chemo alone	47 vs 47	12.8 vs 19.1	1.9 vs 4.0 m	8.4 vs 7.7 m
			PD – (L)1 + Chemotherapy			
Urothelial (1L) IMvigor130 Roche [34]	Atezo + chemo	Chemo alone	447 vs 397	48.1 vs 44.8	8.2 vs 6.3 P=0.007	16.1 vs 13.4 p=0.023 (NS)
Urothelial (1L) KN-361 Merck [35]	Pembro + chemo	Chemo alone	351 vs 352	55 vs 45	8.3 vs 7.1 P=0.0033	17 vs 14.3 P=0.0407
BTC KN-966 Merck [36]	Pembro + chemo	Placebo + Chemo	533 vs 536	29 vs 29	6.5 vs 5.6 P = NS	12.7 vs 10.9 P=0.0034
Endometrial (1L dMMR) KN-868 Merck [37]	Pembro + chemo	Chemo alone	112 vs 113	81.5 vs 70.7 OR: 1.83	NR vs 7.6 P<0.00001	NR vs NR p-=0.0617
Endometrial (1L pMMR) KN-868 Merck [37])	Pembro + chemo	Chemo alone	293 vs 295	70.7 vs 58.1 OR: 1.74	13.1 vs 8.7 P<0.001	NR vs 27.37 P=0.1157
HNSCC (R/M) KN-048 Merck [23]	Pembro + chemo	Cetuximab (EGFRi) + chemo	281 vs 278	36 vs 36	4.9 vs 5.1 p=0.1697	13.0 vs 10.7 p=0.0034
Multiple Myeloma 1L All-comers KN-185 Merck [38]	Pembro + chemo	Chemo alone	149 vs 145	64 vs 62	NR vs NR p=0.75	NR vs NR P=0.97
Gastric/GEJ 1L PD-L1 ≥ 1 or 10 KN-062 Merck [28]	Pembro + chemo	Chemo alone	257 vs 250	49 vs 37	2.0 vs 6.4	12.5 vs 11.1 p=0.05
Multiple Myeloma KN-183 Merck [39]	Pembro + chemo	Chemo alone	125 vs 124	34 vs 40	5.7 vs 7.4 m p=0.98	NR vs 15.2 p=0.95
Non-Sq NSCLC KN-189 Merck [40]	Pembro + chemo	Platinum based chemo	405 vs 202	47.6 vs 18.9	8.8 vs 4.9 p<0.001	NR vs 11.3 p<0.001
Sq. NSCLC 1L All-comers KN-407 Merck [41]	Pembro + chemo	Platinum based chemo	278 vs 281	57.9 vs 28.4	6.4 vs 4.8 m p<0.0001	15.9 vs 11.3 p=0.0017
TNBC 1L PDL1 ≥ 10 KN-355 Merck	Pembro + chemo	Chemo alone	220 vs 103	52.7 vs 40.8	9.7 vs5.6 m p=0.0012	23 vs 16.1 p=0.0093

[42]						
Esophageal 1L<br>All-comers<br>KN-590<br>Merck<br>[43]	Pembro + chemo	Platinum based chemo	373 vs 376	45 vs 29.3	6.3 vs 5.8 m<br>p<0.0001	12.4 vs 9.8<br>p=0.001
			PD – (L)1 + CTL4-A			
Urothelial (1L)<br>DANUBE<br>Astrazeneca<br>[32]	Durva + treme	Chemo	342 vs 344	36 vs 49	3.7 vs 6.7	15.1 vs 12.1<br>p=0.075
Advanced Melanoma<br>CM – 067<br>BMS<br>[20]	Nivo + Ipi	Ipi	314 vs 315	58 vs 19	11.5 vs 2.9	60 vs 19.9
Melanoma (1L)<br>BRAFV600E Wt<br>CM-069<br>BMS<br>[43]	Nivo + Ipi	Ipi alone	72 vs 37	60 vs 11<br>P<0.001	8.9 vs 4.7<br>P<0.002	NR vs 24.8<br>p=0.09
Melanoma (1L)<br>All-comers<br>CM-067<br>BMS<br>[13]	Nivo + Ipi	Ipi	314 vs 315	58 vs 19	11.5 vs 2.9<br>P<0.0001	72.1 vs 19.9<br>P<0.0001
NSCLC 1L<br>PD-L1 ≥ 50%<br>KN-598<br>Merck<br>[44]	Pembro + Ipi	Pembro monoTx	284 vs 284	45.4 vs 45.4	8.2 vs 8.4m<br>; p=0.72	21.4 vs 21.9<br>p=0.74
NSCLC<br>PD-L1 >1%<br>CM-227<br>BMS<br>[17]	Nivo + Ipi	Platinum based chemo	582 vs 583	35.9 vs 30.0	5.1 vs 5.6 m<br>p: Not rep	17.1 vs 14.9<br>p=0.007
Malignant Pleural Mesothelioma<br>CM-743<br>BMS<br>[16]	Nivo + Ipi	Chemo	303 vs 302	40 vs 43	6.8 vs 7.2	18.1 vs 14.1<br>p=0.002
NSCLC 1L<br>All-comers<br>CM-9LA<br>BMS<br>[45]	Nivo + Ipi + chemo	Chemo alone	361 vs 358	38.2 vs 24.9	6.8 vs 5.0<br>P=0.00012	14.1 vs 10.7<br>p=0.0006
			PD – (L)1 + TKI			
Endometrial 2L+<br>KN-775<br>Merck<br>[46]	Pembro + Lenvatinib	Doxorubicin or Paclitaxel	346 vs 351	30 vs 15	6.6 vs 3.8	17.4 vs 12<br>P=0.0001
RCC<br>KN-426<br>Merck<br>[47]	Pembro + axitinib	Sunitinib	432 vs 429	60.4 vs 39.6	15.7 vs 11<br>p<0.0001	45.7 vs 40<br>p=0.001
RCC<br>KN-581<br>Eisai	Pembro + lenvatinib	Sunitinib	355 vs 357	71.0 vs 36.1	23.9 vs 9.2<br>p<0.001	NR vs NR<br>P=0.005

[14]						
HCC<br>LEAP-002<br>Merck<br><br>[48]	Pembro + Lenvatinib	Lenvatinib	395 vs 399	27.8 vs 16.8	8.2 vs 8.0 m<br>p=0.0466	21.2 vs 19<br>p=0.0227
Melanoma<br>LEAP-003<br>Merck<br>[49]	Pembro + Lenvatinib	Pembro monoTx	334 vs 340	40.4 vs 34.1	10.1 vs 4.2	25.8 vs 39.5
NSCLC 1L<br>All-comers; Non-Sq<br>LEAP-006<br>Merck<br>[22]	Pembro + lenvatinib + Plt- chemo	Pembro + plt-chemo	375 vs 373	60.0 vs 53.6	12.2 vs 9.2	21.8 vs 22.1<br>P=0.708
Bladder cancer<br>LEAP-011<br>Merck<br>[50]	Pembro + Lenvatinib	Pembro alone	245 vs 242	33.1 vs 28.9	4.5 vs 4.0 m	11.8 vs 12.9
NSCLC<br>LEAP-008<br>Merck<br>[51]	Pembro + Lenvatinib	Docetaxel	185 vs 189	22.7 vs 14.3	5.6 vs 4.2	11.3 vs 12.0<br>P=0.4342
HNSCC<br>LEAP-10<br>Merck<br>[52]	Pembro + Lenvatinib	Pembro + placebo	256 vs255	46.1 vs 25.4	6.2 vs 2.8<br>P=0.0001	15.0 vs 17.9<br>P=0.882
MSS-CRC<br>LEAP-017<br>Merck<br>[53]	Pembro + Lenvatinib	Rego/<br>TAS-102	241 vs 239	10.4 vs 1.7	3.8 vs 3.3	9.8 vs 9.3 m<br>p=0.0379

Table S2. Summary Statistics for ORR, PFS and OS.

Mean ORR		Mean PFS		Mean OS	
**Experimental**	**Comparator**	**Experimental**	Comparator	Experimental	Comparator
		IO + IO			
42.18%	27.8%	7.8 months	5.7 months	30.25 months	16.78 months
		PD – L (1) Monotherapy			
21.48%	17.38%	3.12 months	4.24 months	12.26 months	9.64 months
		PD – L (1) + Chemotherapy			
48.24%	32.42%	7.94 months	5.6 months	17.9 months	12.58 months
		PD – L (1) + CTLA-4			
50%	23%	11.55 months	5.65 months	72.1 months	22.9 months
		PD – L (1) + TKI			
53.06%	30.83%	15.93 months	9.4 months	33.45 months	29.67 months
		Cumulative Results			
42.5%	26.1%	8.55 months	5.53 months	26.77 months	17.18 months

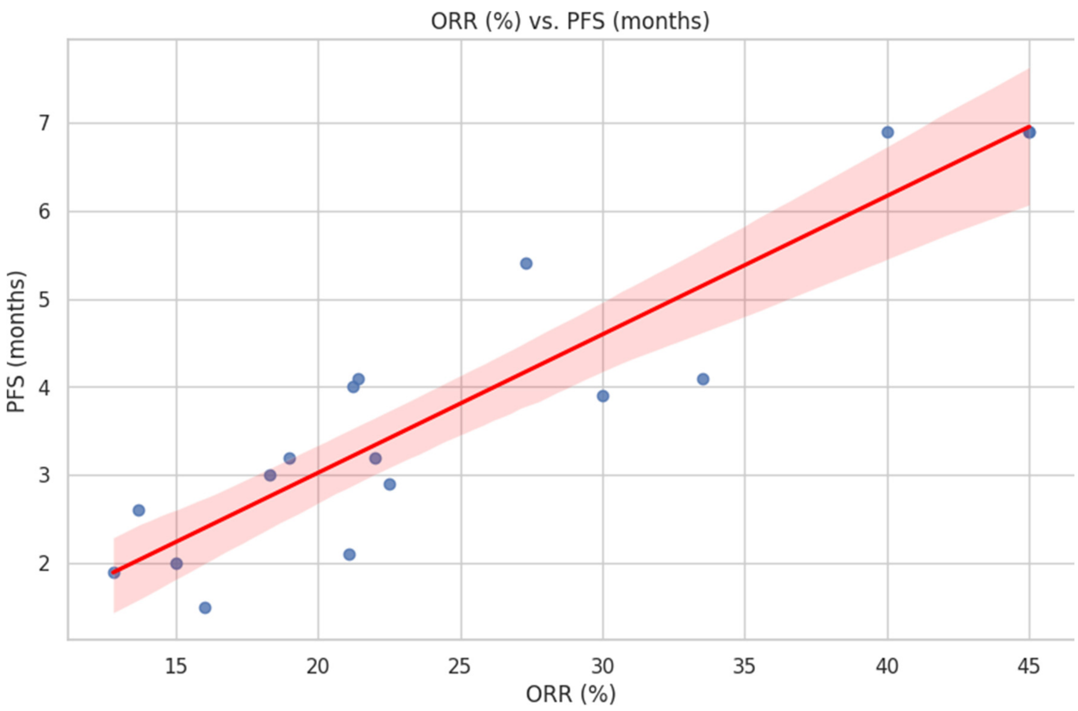

Figure S1. Correlation Between ORR and PFS in Patients Undergoing PD-(L)1 Therapy.

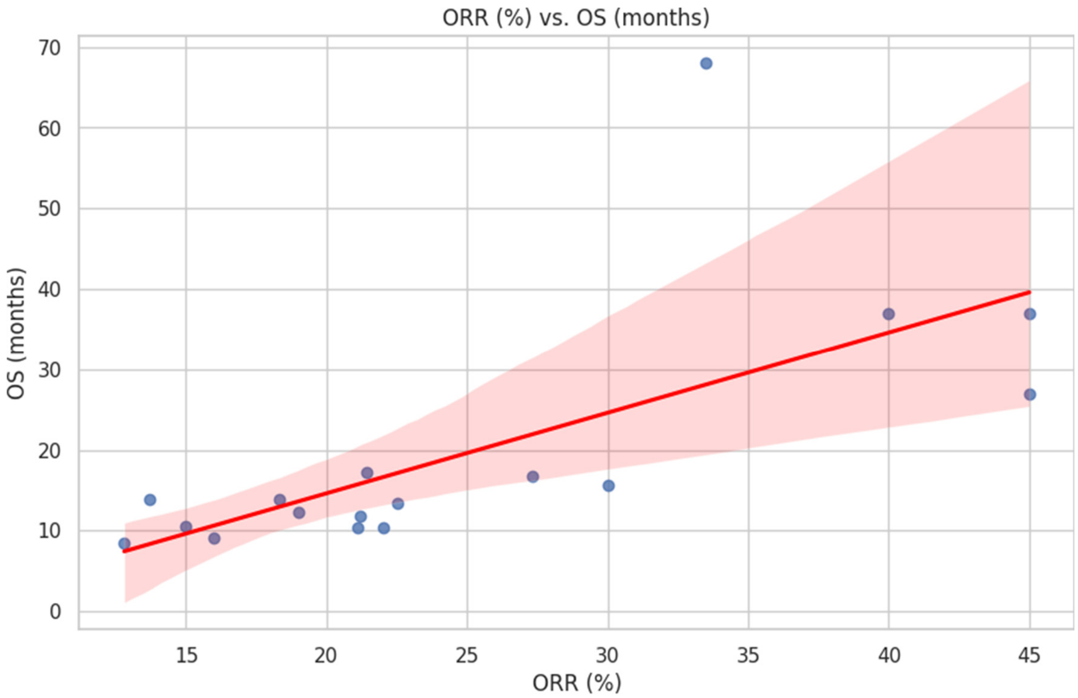

Figure S2. Correlation Between ORR and OS in Patients Undergoing PD-(L)1 Therapy.

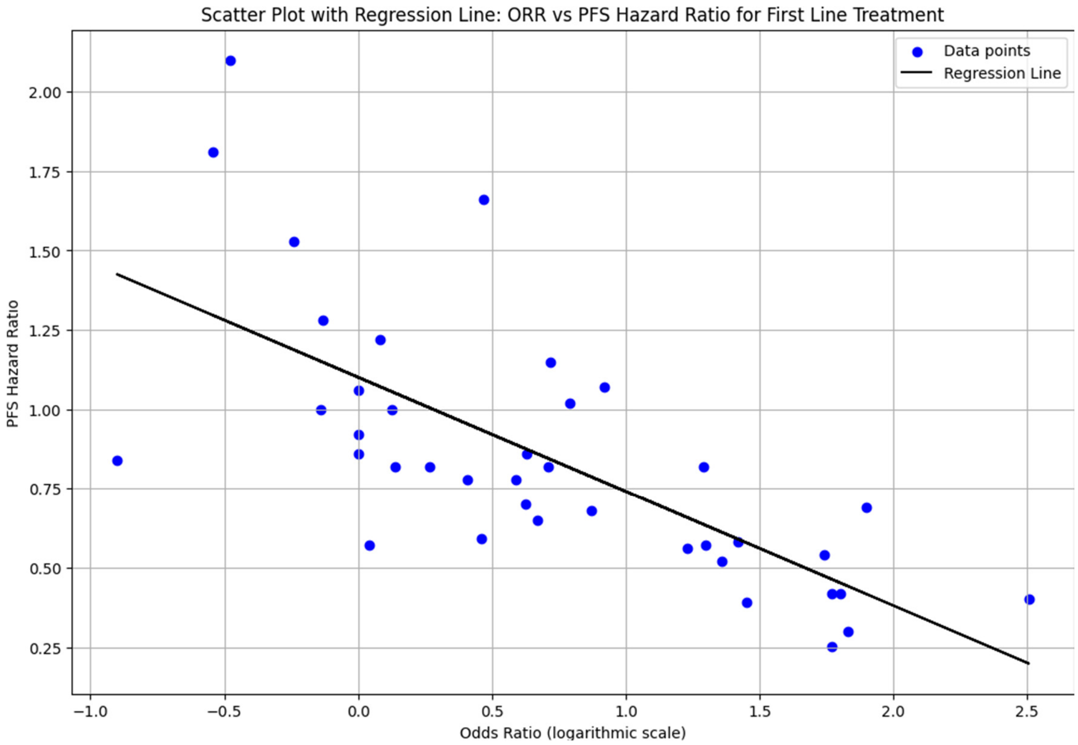

Figure S3. First Line Treatment (PFS).

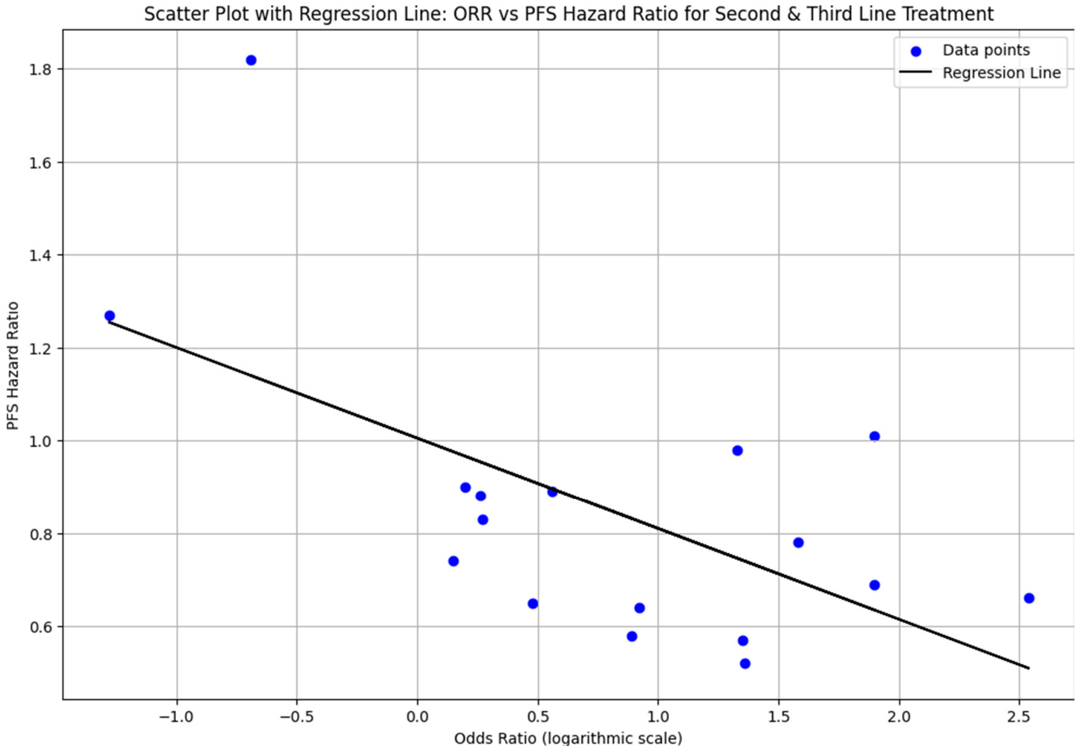

Figure S4. Second & Third Line Treatment (PFS).

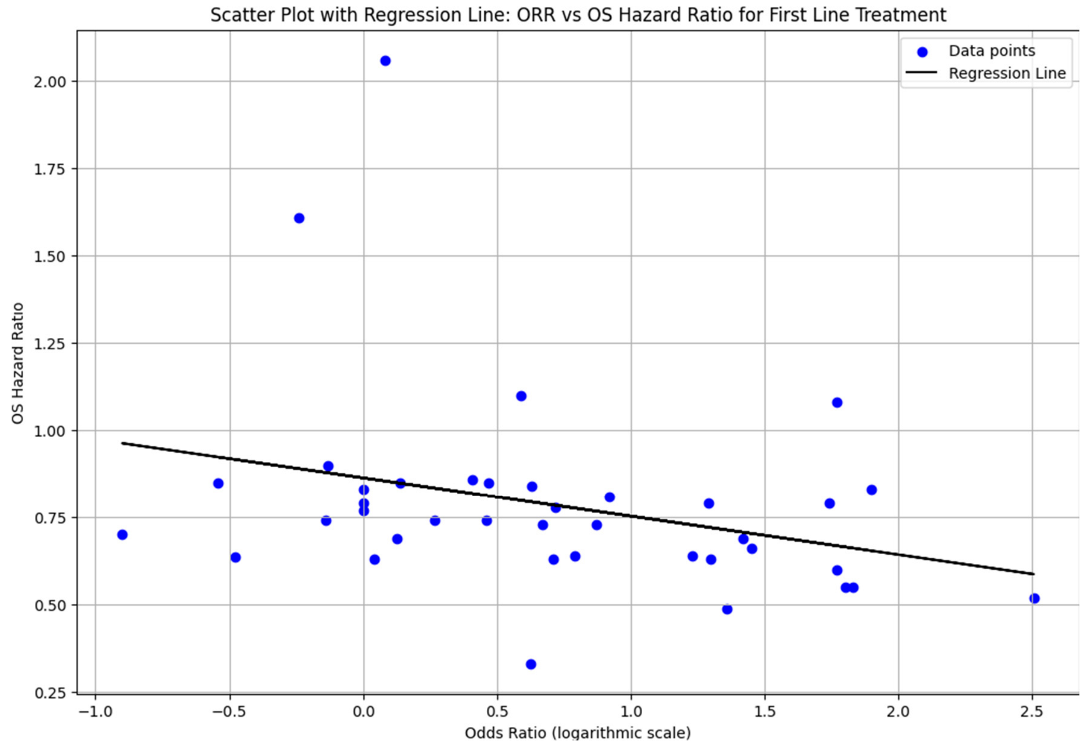

Figure S5. First Line Treatment (OS).

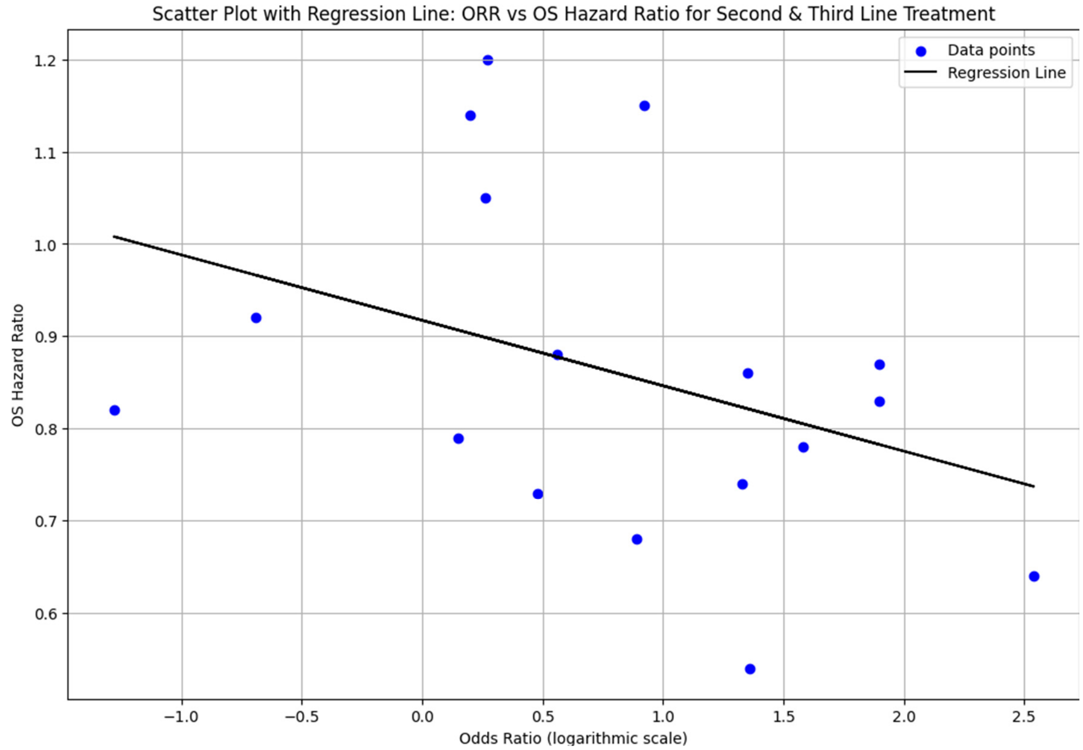

Figure S6. Second and Third Line Treatment (OS).
